# Remote Sensing in Vessel Detection and Navigation

**DOI:** 10.3390/s20205841

**Published:** 2020-10-15

**Authors:** Henning Heiselberg, Andrzej Stateczny

**Affiliations:** 1 National Space Institute, Technical University of Denmark, 2800 Kgs. Lyngby, Denmark; 2 Department of Geodesy, Gdansk University of Technology, 80-233 Gdańsk, Poland; andrzej.stateczny@pg.edu.pl

**Keywords:** vessel detection, navigation, remote sensing

## Abstract

The Special Issue (SI) “Remote Sensing in Vessel Detection and Navigation” highlighted a variety of topics related to remote sensing with navigational sensors. The sequence of articles included in this Special Issue is in line with the latest scientific trends. The latest developments in science, including artificial intelligence, were used. The 15 papers (from 23 submitted) were published.

## 1. Introduction

Earth observation by multispectral, SAR (Synthetic Aperture Radar), and other sensors provides unique global as well as detailed local surveillance. Resolutions allow for vessel detection, classification, and discrimination from, e.g., icebergs and other objects. Important applications include vessel detection and navigation; trafficking and safety; and monitoring the oceans for fishing, oil spills, territorial violations, piracy, refugee boats, emergencies, AIS (Automatic Identification System) spoofing, etc. With global warming, the north-east and -west passages have opened up for shipping, fishing, and cruise ships in uncharted reef-infested territories littered with sea-ice and titanic icebergs.

The Special Issue entitled “Remote Sensing in Vessel Detection and Navigation”, was focused on many aspects of multispectral, multi-sensor, SAR, and other sensors related to science/research, algorithm/technical development, analysis tools, synergy with sensors in multiple wavelengths of the e.m. spectrum, synergy with other measurements such as AIS, as well as reviews of the state-of-the-art in-ocean processes using multispectral and SAR imagery for oceans and sea ice, and vessel monitoring for surveillance, trafficking, and navigation. Topics for the Special Issue included the following:

- Vessel detection, classification, and identification;
- Sea-ice and iceberg detection and tracking;
- Multi-sensor data fusion;
- Autonomous ships navigation;
- Comparative (terrain reference) navigation;
- Change detection for classifying islands, reefs, and other static objects;
- Synergy with and comparison to AIS and other vessel identification data;
- Synergies between satellite sensors with airborne platforms; multiple satellite SAR; optical and thermal infrared sensors including finer resolution sensors, for example, sentinels and other satellites, and in situ measurements;
- The use of multispectral, multiple frequencies, and polarizations to interpret and quantitatively assess various ocean surfaces, currents, and sea ice phenomena for navigation;
- Interferometric and Doppler-derived SAR oceanic and sea ice applications focused on surface motion;
- Validation studies for vessel, ocean, and sea-ice parameters based on in situ and airborne data collections;
- Use of machine learning and the build-up of annotated training databases;
- Artificial Intelligence for image data processing.

In this article we provide a brief overview of the published papers, in particular the use of advanced modern technologies and data fusion techniques. These two areas seem to be the right direction for the future development of vessel detection and navigation.

## 2. Overview of Contributions

### 2.1. Convolutional Neural Networks for Detection and Classification of Vessels

Convolutional Neural Networks (CNN) are state of the art machine learning algorithms for detection and classification of objects in images. This Special Issue includes several works on CNN applied SAR images of vessels.

Dai et al. [1] propose a novel SAR ship detector, based on three subnetworks, which is designed especially for small ships in a noisy background. Using the public SAR ship dataset (SSDD) and China Gaofen-3 satellite SAR images, their method shows excellent performance for detecting the multiscale and small-size ships with respect to some competitive models and exhibits high potential in practical application.

Fu et al. [2] investigate very deep neural networks and the problem of saturation that limits the recognition accuracy. They incorporate micro convolutional modules, residual structures and other improvements. Testing their model on a simulated dataset of HRRP signals obtained from thirteen 3D CAD object models, their model is capable of achieving higher recognition accuracy and robustness than other common network structures.

Pan et al. [3] propose a new method for dealing with the arbitrary rotations of ships in noisy SAR images by removing redundancy and improving detection. A multi-stage rotational region-based network (MSR2N) is proposed which contains three modules and several other characteristics. MSR2N is more suitable and robust for ship detection in SAR images. The experimental results on their SAR ship detection dataset (SSDD) show that the MSR2N has achieved state-of-the-art performance.

### 2.2. Vessel Detections by Radar

Radars are historically the most common method for navigation and vessel detection. There are, however, certain limitations which are addressed by two papers in this Special Issue.

Jiang et al. [4] address the problem of R-mode AIS signals where a ship may only receive signals from one shore station. Lacking range information it cannot determine its position. They describe a novel method using several ship antennas which then can determine its position by triangulation and discuss the precision of this method. Their proposed method expands the application scope of the AIS R-mode positioning system widely.

Lisowski [5] address the problem of inaccurate and limited radar data for autonomous ships during maneuvering. They analyze the use of game theory for decision processes in order to predict the safest trajectories, and do simulation studies of a positional game algorithm. A real situation was studied as an example, where a vessel had to sail among nine other ships. The sensitivity characteristics of safe trajectories under conditions of both good and limited visibility at sea were presented.

### 2.3. Vessel Detections by Video Sensors

Video surveillance is becoming increasingly popular in the process of detecting ships at short distances. It is particularly useful in lintel systems both on sea and inland waterways. There are also articles devoted to this issue in the SI.

Wawrzyniak et al. [6] present a method that allows for the detection and tracking of ships using the video streams of existing monitoring systems for ports and rivers. The article presents the way and results of experiments conducted on selected groups of data using selected cameras with different working parameters. Experiments were carried out in different locations, variable lighting and weather conditions. The detection targets were variable vessels. The obtained results confirmed the correctness of the adopted solution, although minor problems were encountered in difficult weather conditions.

Polap and Wlodarczyk-Sielicka [7] address the problem of vessel classification, not by classifying whole input data, but smaller parts of the data. A duplicate way is to divide the image of the ship into smaller parts and classify them into vectors that can be identified as features by means of a convolutional neural network (CNN). The concept presented in the article reflects the title mechanism of the word sack, where the created feature vectors can be called words, and with their help the solution can assign images a specific class. The results of two tests are presented, in which, first, two classes were analyzed, and second, the authors used much larger sets of images belonging to five types of ships. The method described in the paper allowed to obtain better results by about 5% every year, which is good for future research. The given solution may be an alternative approach to the classification of unconventional ships. 

### 2.4. Object Detections by Different Sensors

A number of articles are devoted to the processing of data from various other sensors in the process of detection and eventual identification of floating objects.

Ru et al. [8] present a detection and tracking algorithm based on the Gibbs Generalized Labeled Multi-Bernoulli (GLMB) filter for estimating recognizable target groups. Once the state of a group target has been estimated, we extract relevant information from the estimation data to assess whether the structure or state of concentration of the target group has changed. Finally, they conduct several experiments to verify the algorithm.

Shan et al. [9] address the problem of accurate detection of the coastline and nearby ships with the available sensors due to the complex environment and six degrees of freedom of movement. The article combines data from cameras and inertial sensors and proposes an innovative algorithm for detecting sea targets based on camera position in relation to movement. The developed algorithm consists of semiconductor lighting estimation, semiconductor lighting detection and target weakness detection. Experimental results have been compared to the results of the experiments indicating that the proposed algorithm achieves a higher accuracy of detection near ships and may be of significant importance in the development of unmanned ships. 

Willburger et al. [10] demonstrate of the project AMARO (Autonomous Real-Time Detection of Moving Maritime Objects) running at DLR. AMARO is a feasibility study of an on-board ship detection system involving on-board processing and real-time communication. The article describes the scope, purpose and design of the AMARO system, as well as detailed results of the flight experiment. The operation of the prototype system was successfully demonstrated on an on-board platform in spring 2018. Ground users could be informed about the detected ships within minutes after their detection without a direct communication link.

Dong et al. [11] address the problem of passive sonar detection and identification in low signal to noise rate conditions. They proposed adaptive intrawell matched stochastic resonance Adaptive Linear Amplifiers (AIMSR) method, aiming to break through the limitation of the conventional adaptive line enhancers (ALE) method by nonlinear filtering effects. The verification of the application was carried out experimentally in a tank with an autonomous submarine vehicle (AUV) in order to confirm the feasibility and effectiveness of the proposed method. The results indicate that the proposed method is superior to the conventional adaptive line enhancers (ALE) method.

### 2.5. Multispectral Techniques for Remote Sensing

The optical spectrum was also investigated in two papers, where special spectrum sensor features could be exploited.

Kim et al. [12] propose a new method for ground-based ship detection exploiting the strong CO_2_ absorption in the mid-infrared band. Since ship emissions are hot, their CO_2_ emission spectrum is broader than the subsequent absorption band in the colder atmosphere. As a result, a double peak remains in the IR spectrum that allow for robust remote detection of ships in the mid-IR band.

Heiselberg [13] demonstrated for the first time that the time delay between recording the 13 images for the Sentinel-2 multispectral imager can be exploited to determine ship and aircraft velocities from space. The movement in the image during recording is substantial for aircrafts but only just measurable for the much slower ships. The velocities (and altitudes) can be accurately determined for aircrafts and reasonably for ships without wakes. When strong wakes are visible, they distort the spectrum spatially, which can partially be corrected for.

### 2.6. Autonomous Ships’ Navigation

The next two articles concern unmanned surface vehicles for hydrographic tasks, which have been developing rapidly in recent years pushing out more and more manned ships.

Specht et al. [14] address the problem of steering precision on low-cost Unmanned Surface Vessel (USV) during automatic survey. One of the challenges of modern hydrography are measurements in shallow waters less than 1 m deep. A potential solution seems to be the use of USV of low draught as an alternative method for such bathymetric measurements. The article presents the modernization of USV allowing for the realization of automatic mode in low-budget USV. It presents an evaluation of the usefulness of the popular autopilot and cooperating with its cheap satellite navigation system (GNSS) receiver in the automatic process of bathymetric measurements. The results obtained in terms of distance from the planned trajectory were evaluated statistically. 

Stateczny et al. [15] address the issue of precise navigation of the hydrographic unit on the measuring profile with the use of a nonlinear adaptive autopilot. Precise navigation in relation to the measurement profile avoids registration of unnecessary data and saves time and costs of hydrographic measurements both during the registration process and during data processing. This article discusses the results of experiments concerning hydrographic measurements performed in real conditions with a new generation HydroDron vehicle.

## 3. Conclusions

The Special Issue entitled “Remote Sensing in Vessel Detection and Navigation” comprised 15 articles on many topics related to remote sensing with navigational sensors. In this paper, we have presented short introductions of the published articles.

It can be said that navigation and vessel detection still remain important and hot topics, and a lot of work will continue to be done worldwide. New techniques and methods for analyzing and extracting information from navigational sensors and data have been proposed and verified. Some of these will provoke further research, and some are already mature and can be considered for industrial implementation and development.
